# Machine Learning Applications in Head and Neck Radiation Oncology: Lessons From Open-Source Radiomics Challenges

**DOI:** 10.3389/fonc.2018.00294

**Published:** 2018-08-17

**Authors:** Hesham Elhalawani, Timothy A. Lin, Stefania Volpe, Abdallah S. R. Mohamed, Aubrey L. White, James Zafereo, Andrew J. Wong, Joel E. Berends, Shady AboHashem, Bowman Williams, Jeremy M. Aymard, Aasheesh Kanwar, Subha Perni, Crosby D. Rock, Luke Cooksey, Shauna Campbell, Pei Yang, Khahn Nguyen, Rachel B. Ger, Carlos E. Cardenas, Xenia J. Fave, Carlo Sansone, Gabriele Piantadosi, Stefano Marrone, Rongjie Liu, Chao Huang, Kaixian Yu, Tengfei Li, Yang Yu, Youyi Zhang, Hongtu Zhu, Jeffrey S. Morris, Veerabhadran Baladandayuthapani, John W. Shumway, Alakonanda Ghosh, Andrei Pöhlmann, Hady A. Phoulady, Vibhas Goyal, Guadalupe Canahuate, G. Elisabeta Marai, David Vock, Stephen Y. Lai, Dennis S. Mackin, Laurence E. Court, John Freymann, Keyvan Farahani, Jayashree Kaplathy-Cramer, Clifton D. Fuller

**Affiliations:** ^1^Department of Radiation Oncology, University of Texas MD Anderson Cancer Center, Houston, TX, United States; ^2^Baylor College of Medicine, Houston, TX, United States; ^3^Università degli Studi di Milano, Milan, Italy; ^4^Department of Clinical Oncology and Nuclear Medicine, Alexandria University, Alexandria, Egypt; ^5^McGovern Medical School, University of Texas, Houston, TX, United States; ^6^School of Medicine, The University of Texas Health Science Center San Antonio, San Antonio, TX, United States; ^7^Department of Cardiology, Massachusetts General Hospital, Harvard Medical School, Boston, MA, United States; ^8^Furman University, Greenville, SC, United States; ^9^Abilene Christian University, Abilene, TX, United States; ^10^Department of Radiation Oncology, Oregon Health and Science University, Portland, OR, United States; ^11^Department of Radiation Oncology, Memorial Sloan Kettering Cancer Center, New York, NY, United States; ^12^Texas Tech University Health Sciences Center El Paso, El Paso, TX, United States; ^13^University of North Texas Health Science Center, Fort Worth, TX, United States; ^14^Department of Radiation Oncology, Cleveland Clinic, Cleveland, OH, United States; ^15^Colgate University, Hamilton City, CA, United States; ^16^Graduate School of Biomedical Sciences, MD Anderson Cancer Center, Houston, TX, United States; ^17^Department of Radiation Physics, Graduate School of Biomedical Sciences, MD Anderson Cancer Center, Houston, TX, United States; ^18^Moores Cancer Center, University of California, La Jolla, San Diego, CA, United States; ^19^Dipartimento di Ingegneria Elettrica e delle Tecnologie dell'Informazione, Università Degli Studi di Napoli Federico II, Naples, Italy; ^20^Department of Biostatistics, University of Texas MD Anderson Cancer Center, Houston, TX, United States; ^21^Fraunhofer-Institut für Fabrikbetrieb und Automatisierung (IFF), Magdeburg, Germany; ^22^Department of Computer Science, University of Southern Maine, Portland, OR, United States; ^23^Indian Institute of Technology Hyderabad, Sangareddy, India; ^24^University of Iowa, Iowa City, IA, United States; ^25^University of Illinois at Chicago, Chicago, IL, United States; ^26^Department of Biostatistics, School of Public Health, University of Minnesota, Minneapolis, MN, United States; ^27^Department of Head and Neck Surgery, University of Texas MD Anderson Cancer Center, Houston, TX, United States; ^28^Frederick National Laboratory for Cancer Research, Leidos Biomedical Research, Inc., Frederick, MD, United States; ^29^National Cancer Institute, Rockville, MD, United States; ^30^The Russell H. Morgan Department of Radiology and Radiological Science, Johns Hopkins Medicine, Baltimore, MD, United States; ^31^Department of Radiology and Athinoula A. Martinos Center for Biomedical Imaging, MGH/Harvard Medical School, Boston, MA, United States

**Keywords:** machine learning, radiomics challenge, radiation oncology, head and neck, big data

## Abstract

Radiomics leverages existing image datasets to provide non-visible data extraction via image post-processing, with the aim of identifying prognostic, and predictive imaging features at a sub-region of interest level. However, the application of radiomics is hampered by several challenges such as lack of image acquisition/analysis method standardization, impeding generalizability. As of yet, radiomics remains intriguing, but not clinically validated. We aimed to test the feasibility of a non-custom-constructed platform for disseminating existing large, standardized databases across institutions for promoting radiomics studies. Hence, University of Texas MD Anderson Cancer Center organized two public radiomics challenges in head and neck radiation oncology domain. This was done in conjunction with MICCAI 2016 satellite symposium using Kaggle-in-Class, a machine-learning and predictive analytics platform. We drew on clinical data matched to radiomics data derived from diagnostic contrast-enhanced computed tomography (CECT) images in a dataset of 315 patients with oropharyngeal cancer. Contestants were tasked to develop models for (i) classifying patients according to their human papillomavirus status, or (ii) predicting local tumor recurrence, following radiotherapy. Data were split into training, and test sets. Seventeen teams from various professional domains participated in one or both of the challenges. This review paper was based on the contestants' feedback; provided by 8 contestants only (47%). Six contestants (75%) incorporated extracted radiomics features into their predictive model building, either alone (*n* = 5; 62.5%), as was the case with the winner of the “HPV” challenge, or in conjunction with matched clinical attributes (*n* = 2; 25%). Only 23% of contestants, notably, including the winner of the “local recurrence” challenge, built their model relying solely on clinical data. In addition to the value of the integration of machine learning into clinical decision-making, our experience sheds light on challenges in sharing and directing existing datasets toward clinical applications of radiomics, including hyper-dimensionality of the clinical/imaging data attributes. Our experience may help guide researchers to create a framework for sharing and reuse of already published data that we believe will ultimately accelerate the pace of clinical applications of radiomics; both in challenge or clinical settings.

## Introduction

Radiomics, or texture analysis, is a rapidly growing field that extracts quantitative data from imaging scans to investigate spatial and temporal characteristics of tumors (1). To date, radiomics feature signatures have been proposed as imaging biomarkers with predictive and prognostic capabilities in several types of cancer (2–6). Nevertheless, non-uniformity in imaging acquisition parameters, volume of interest (VOI) segmentation, and radiomics feature extraction software tools make comparison between studies difficult, and highlight unmet needs in radiomics (7). Specifically, reproducibility of results is a necessary step toward validation and testing in real-world multicenter clinical trials (8). Another commonly emphasized bias of high-throughput classifiers such as those in radiomics is the “curse of dimensionality,” which stems from having relatively small datasets and a massive number of possible descriptors (9).

Multi-institutional cooperation and data sharing in radiomics challenges can address, in particular, the issue of dimensionality and advance the field of quantitative imaging (10, 11). Hence, the Quantitative Imaging Network (QIN) of the National Cancer Institute (NCI) (12) started the “Challenges Task Force” with singular commitment to collaborative projects and challenges that leverage analytical assessment of imaging technologies and quantitative imaging biomarkers (13). To this end, and at the request of NCI and invitation from Medical Image Computing and Computer Assisted Intervention [MICCAI] Society, the head and neck radiation oncology group at The University of Texas MD Anderson Cancer Center organized two radiomics competitions. Oropharyngeal cancer (OPC) was chosen as a clinically relevant realm for radiomics hypothesis testing. Using manually-segmented contrast-enhanced computed tomography (CECT) images and matched clinical data, contestants were tasked with building one of 2 models. These included: (i) a classification model of human papillomavirus (HPV) status; and (ii) a predictive model of local tumor recurrence, following intensity-modulated radiation treatment (IMRT) (14).

We had several motivations for organizing these radiomics challenges. First: To demonstrate that radiomics challenges with potential clinical implementations could be undertaken for MICCAI. Second: To identify whether Kaggle in Class, a commercial educationally-oriented platform could be used as an avenue to make challenges feasible in the absence of custom-constructed websites or elaborate manpower. The main aim of this review is to detail the mechanics and outcomes of our experience of using a large standardized database for radiomics machine-learning challenges. We previously detailed the data included in both our challenges in a recently published data descriptor (14). Here, we will continue to outline the “challenge within a challenge” to provide a template workflow for initiating substantial platforms for facilitating “multi-user” radiomics endeavors. By pinpointing these hurdles, we hope to generate insights that could be used to improve the design and execution of future radiomics challenges as well as sharing of already published radiomics data in a time-effective fashion.

## Materials and methods for challenges

At the invitation of NCI and MICCAI, the head and neck radiation oncology group at The University of Texas MD Anderson Cancer Center organized two public head and neck radiomics challenges in conjunction with the MICCAI 2016: Computational Precision Medicine satellite symposium, held in Athens, Greece. Contestants with machine-learning expertise were invited to construct predictive models based on radiomics and/or clinical data from 315 OPC patients to make clinically relevant predictions in the head and neck radiation oncology sphere.

### Database

After an institutional review board approval, diagnostic CECT DICOM files and matched clinical data were retrieved for OPC patients who received curative-intent IMRT at our institution between 2005 and 2012 with a minimum follow-up duration of 2 years. A key inclusion criterion was pre-treatment testing for p16 expression as a surrogate for HPV status. 315 patients with histopathologically-proven OPC were retrospectively restored from our in-house electronic medical record system, ClinicStation. The study was Health Insurance Portability and Accountability Act (HIPAA) compliant, and the pre-condition for signed informed consent was waived (15).

We then imported contrast-enhanced CT scans of intact tumor that were performed not only before the start of IMRT course but also before any significant tumor volume-changing procedures, i.e., local or systemic therapies. Although all patients were treated at the same institute, their baseline CECT scans were not necessarily obtained from the same scanner, i.e., different scanners within the same institute or less commonly baseline scans from outside institute. Hence, thorough details of images characteristics and acquisition parameters were kept in the DICOM header and made available as a Supplementary Table. A publicly available anonymizer toolbox, DICOM Anonymizer version 1.1.6.1, was employed to anonymize protected health information (PHI) on all DICOM files in accordance with the HIPAA, as designated by the DICOM standards from the Attribute Confidentiality Profile (DICOM PS 3.15: Appendix E) (16).

The selected CT scans were imported to VelocityAI 3.0.1 software (powered by VelocityGrid), which was used by two expert radiation oncologists to segment our VOIs in a slice-by-slice fashion. VOIs were defined as the pre-treatment gross tumor volume (GTV) of the primary disease (GTVp), which was also selected as the standardized nomenclature term. Gross nodal tumor volumes also were segmented to provide a complete imaging dataset that can benefit other radiomics studies in the head and neck cancer domain. However, contestants were clearly instructed to include only GTVp in regions of interest for robust texture analysis.

Segmented structures in congruence with matched clinical data constituted the predictor variables for both challenges. Clinical data elements comprised patient, disease, and treatment attributes that are of established prognostic value for OPC (17). A matching data dictionary of concise definitions, along with possible levels for each clinical data attribute, was provided to contestants as a “ReadMe” CSV file (Table 1).

**Table 1:** Supplemental information about data provided for radiomics challenges.

**Data element**	**Description**
Patient ID	Numbers given randomly to the patient after anonymization of the DICOM protected health identifier (PHI) tag (0010,0020) that corresponds to medical record number
HPV/p16 status	HPV status, as assessed by HPV DNA *in situ* hybridization (57) and/or p16 protein expression via immunohistochemistry, with the results described as 1 (i.e., positive) or 0 (i.e., negative)
Gender	Patient's sex
Age at diagnosis	Patient's age in years at the time of diagnosis
Race	American Indian/Alaska Native, Asian, Black, Hispanic, White, or not applicable
Tumor laterality	Right, left, or bilateral
Oropharynx subsite of origin	Subsite of the tumor within the oropharynx, i.e., base of tongue (21) or tonsil/soft palate/pharyngeal wall/glossopharyngeal sulcus/other (no single subsite of origin could be identified)
T category	Description of the original (primary) tumor with regard to size and extent per the American Joint Committee on Cancer (AJCC) and Union for International Cancer Control (UICC) cancer staging system, i.e., T1, T2, T3, or T4 (https://cancerstaging.org/references-tools/Pages/What-is-Cancer-Staging.aspx)
N category	Description of whether the cancer has reached nearby lymph nodes, per the AJCC and UICC cancer staging system, i.e., N0, N1, N2a, N2b, N2c, or N3 (https://cancerstaging.org/references-tools/Pages/What-is-Cancer-Staging.aspx)
AJCC stage	AJCC cancer stage (https://cancerstaging.org/references-tools/Pages/What-is-Cancer-Staging.aspx)
Pathologic grade	Grade of tumor differentiation, i.e., I, II, III, IV, I-II, II-III, or not assessable
Smoking status at diagnosis	Never, current, or former smoker
Smoking pack-years	An equivalent numerical value of lifetime tobacco exposure; 1 pack-year is defined as 20 cigarettes smoked every day for 1 year

We also provided contestants with a list of suggested open-source infrastructure software that supports common radiomics workflow tasks such as image data import and review as well as radiomics feature computation, along with links to download the software. After completion of the challenge, a complete digital repository was deposited (figshare: https://doi.org/10.6084/m9.figshare.c.3757403.v1 and https://doi.org/10.6084/m9.figshare.c.3757385.v1) (18, 19) and registered as a public access data descriptor (14).

### Challenge components

Challenge components were identified as a function of the hosting platform.

#### Hosting platform

**Figure 1:**
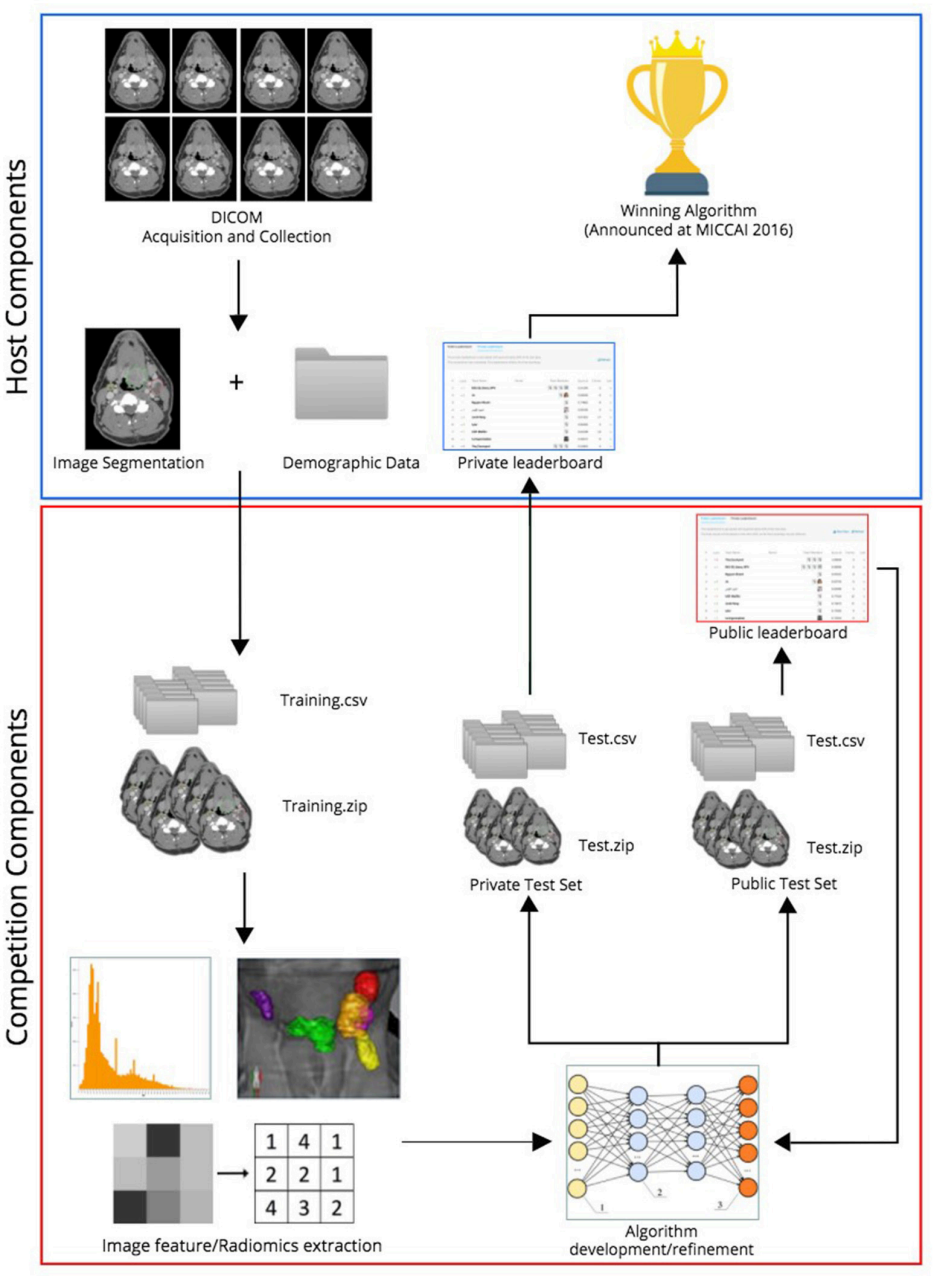
Workflow of radiomics challenges.

In the two radiomics challenges, organized on Kaggle-in-Class, contestants were directed to construct predictive models that (i) most accurately classified patients as HPV positive or negative compared with their histopathologic classification (http://inclass.kaggle.com/c/oropharynx-radiomics-hpv), and (ii) best predicted local tumor recurrence (https://inclass.kaggle.com/c/opc-recurrence). Kaggle-in-Class (https://inclass.kaggle.com/) is a cloud-based platform for predictive modeling and analytics contests on which researchers post their data and data miners worldwide attempt to develop the most optimal predictive models. The overall challenge workflow is portrayed in Figure 1.

Anonymized imaging and clinical data belonging to the cohort of 315 OPC patients were uploaded to the Kaggle in Class server almost evenly split between the training subset and test subset, encompassing 150 and 165 patients, respectively, in separate CSV files and DICOM folders. Subjects were randomly assigned to either training or test sets via random number generation. Caution was taken to make outcome of interest (HPV status for the first challenge and local control for the second one) proportionally distributed in training and test sets. For the test set, contestants were blinded to the outcome.

#### Evaluation metric

The evaluation metric for both competitions was area under the receiver operating characteristic curve (AUC) of the binary outcomes, i.e., “positive” vs. “negative” for the “HPV” challenge or “recurrence” vs. “no recurrence” for the “local recurrence” challenge.

#### Scoring system

Kaggle-in-Class further splits the test set randomly into two subsets of approximately equal size again with outcome of interest equally distributed. One subset was made public to contestants, named the “Public Test subset.” The other subset was held out from the contestants, with only challenge organizers having access to it, named the “Private Test subset.” The performance of the contestants' models was first assessed on the public test set and results were posted to a “Public leaderboard.” The public leaderboards were updated continuously as contestants made new submissions, providing real-time feedback to contestants on the performance of their models on the public test subset relative to that of other contestants' models.

**Figure 2:**
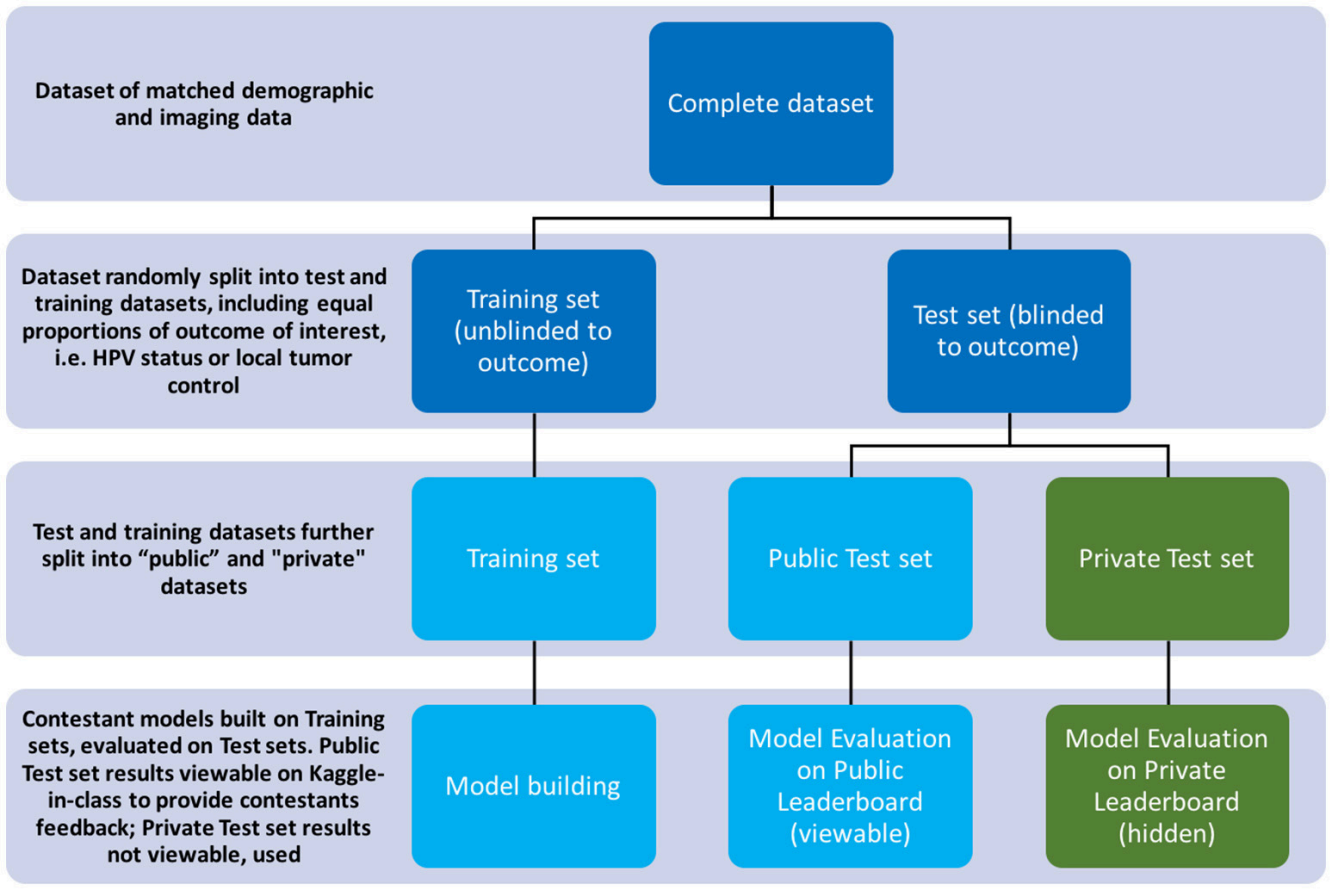
Diagram illustrating the splitting of datasets per the challenge's rules.

The private leaderboard was accessible only to the organizers of the challenges. Toward the end of the challenge, each contestant/team was allowed to select his/her/their own two “optimal” final submissions of choice. Contestants were then judged according to the performance of their chosen model(s) on the private test subset, according to the private leaderboard. The contestant/team that topped the “private leaderboard” for each challenge was declared the winner of the challenge. The distinction between training/test set and public/private subset terminology is further illustrated in Figure 2.

#### Challenges rules

Teams were limited to a maximum of two result submissions per team per day. There was no maximum team size, but merging with or privately sharing code and data with other teams was prohibited.

#### Challenges organizers-contestants interaction

To enable contestants to communicate with the organizing committee, the e-mail address of one of the organizers was made available on the Kaggle in Class and MICCAI websites. Also, the organizers created and closely followed a discussion board where updates or topics of common interest were publicly shared. After announcing the winners, questionnaires were distributed to contestants to get their feedback, which greatly contributed to this review paper.

## Challenge results

Seventeen teams participated in either one or both challenges, accounting for a total of 23 enrollments. The “HPV” challenge recorded nine enrollments comprising three multiple-member teams and six individual contestants. The “local recurrence” challenge, on the other hand, had four multiple-member teams and 10 individual contestants. The following results are derived from the questionnaires, which were filled out by eight teams. Detailed responses of contestants to post-challenges surveys are tabulated in Supplementary Table 1. Contestants came from various professional domains, e.g., biostatistics, computer science, engineering, medical physics, mathematics, and radiation oncology. The dedicated time per participant for each challenge ranged from 6 to 30 h. Teams included as many as seven members with the same or different institutional affiliations.

The data analytical algorithms showed wide variation in methods and implementation strategies. The programming platforms used to extract quantitative radiomics features included MATLAB, R, and Python. Most contestants (63%) developed their own scripts to extract radiomics features. The Imaging Biomarker Explorer (IBEX) software, developed by the Department of Radiation Physics at MD Anderson (20), was the second most commonly used software among the other contestants (38%). The machine-learning techniques used included random forest with class balancing, logistic regression with gradient descent or extreme gradient boosting trees, least absolute shrinkage, and selection operator (Lasso) regression, and neural networks. Interestingly, one contestant reported applying an ensemble combination of classifiers, including random forests, a naïve Bayes classifier, and Association for Computing Machinery classifiers, as well as boosting algorithms, including AdaBoost, and oversampling techniques, including Synthetic Minority Over-sampling Technique. The most commonly used statistical tests included leave-one-out cross-validation, the Wilcoxon rank-sum test, and sparse matrices.

The key, relevant radiomics features selected by these various machine-learning algorithms encompassed various first- and second-order features. The chosen first-order features included the “intensity” feature of maximum intensity and the “shape” features of primary tumor volume, longest and shortest radii, and Euclidean distance (in mm, with respect to centroids) between the primary tumor and the lymph nodes (minimum, maximum, mean, and standard deviation). The chosen second-order features included gray-level co-occurrence matrix and local binary pattern.

Key clinical data commonly selected and modeled by contestants included smoking pack-years, T category, N category, and tumor subsite of origin, e.g., tonsil or base of tongue. Most contestants (77%) incorporated extracted radiomics features into their model, either alone (62%), as was the case with the winning team of the “HPV” challenge, or in conjunction with matched clinical attributes (16%). Meanwhile, only 23% of contestants built their models relying solely on clinical data, including the winner of the “local recurrence” challenge.

Per contestant feedback, the obstacles to developing sound machine-learning predictive models were largely technical in nature. Fifty percent of questionnaire respondents reported inability to extract radiomics features, especially global directional features, for some images. This was the leading cause of missing values, which were difficult to handle for most contestants. Other barriers involved segmentation issues where some VOIs—according to one contestant—were not consistently named across the whole cohort. A few contestants also reported that some GTVp contours did not adequately represent the primary tumor lesions, i.e., some slices within the VOI were not segmented, or GTVp contours were totally absent. In some cases, only metastatic lymph nodes (i.e., gross nodal tumor volume) were segmented, per one contestant. Nonetheless, all but one team expressed enthusiasm toward participating in future machine-learning challenges.

For the “HPV” challenge, the winners were a team of academic biostatisticians with a radiomics-only model that achieved an AUC of 0.92 in the held-out, private test subset. Their feature selection approach yielded the “shape” features of “mean breadth” and “spherical disproportion” as most predictive of HPV status, suggesting that HPV-associated tumors tend to be smaller and more homogeneous. On the other hand, the winner of the “local recurrence” challenge was a mathematics/statistics college student who exclusively used clinical features to build a model that achieved an AUC of 0.92 in the private test subset. The AUCs of all contestants' models and their corresponding final ranking in the private leaderboard are provided in Supplementary Tables 2, 3.

The winner of each challenge was invited to share their approach and models via video conference at the Computational Precision Medicine satellite workshop as part of the MICCAI 2016 program that took place in Athens, Greece. Moreover, each winner was offered a manuscript acceptance (after editorial review) with fees waived to describe their approach and algorithm in an international, open-access, peer-reviewed journal sponsored by the European Society for Radiotherapy and Oncology. The winners of the “HPV” challenge recently reported their approach in designing a statistical framework to analyze CT images to predict HPV status (21).

## Discussion

The process of designing and executing the radiomics challenge was inevitably filled with difficult decisions and unexpected issues, from which we have yielded numerous insights. We have enumerated these challenges and derived lessons in Table 2.

**Table 2:** Challenges and derived lessons from organizing open-source radiomics challenges.

**Challenges**
• Paucity of open-source freely available radiomics datasets • Establishing database: size vs. time • Data anonymization • Quality assurance: before, during, and after the challenge • Understanding contestants' preferences • Clarity of challenge rules verbiage • Hyperdimensionality of radiomics co-variates and subsequent overtraining • Low post-challenge survey response rate • Discrete scanners, acquisition parameters, and segmentation techniques
**Derived Lessons**
• Use common ontology guidelines to assign nomenclature for target volumes and clinical data • Use efficient, secure solution such as RSNA CTP to minimize time/resource burden • Test run data prior to start of radiomics challenge to identify additional issues
• Adopt “Public/Private leaderboard” challenges to mitigate overtraining/overfitting • Choice of data type and sources (i.e., single vs. multi-institutional) depends on specific aims of radiomics challenge • Provide contestants multiple ways to analyze data whenever possible, e.g., with/without artifacts to account for variation in contestants' preferences • Rules must be clear and consistent with all other aspects of challenge design • Proper incentives built into the radiomics challenge encourage participation and subsequent feedback • Post-challenge permanent data repository and descriptor

### “Challenge within a challenge” and derived lessons

Before, during and even after the radiomics challenges, we encountered situations which provided us insight into improving future radiomics challenges. We will now detail learning points derived from our experience.

#### Database size

The usefulness of a database for radiomics analysis increases as more and more cases are added. However, limits on time, personnel, and available patient data place constraints on database collection and thus the ability to yield insights from radiomics analysis. Also relevant to imaging data collection is the variation in imaging acquisition parameters and disease states within a disease cohort. In our case, as in many practical classification problems, HPV status and local control rates following IMRT for OPC patients tend to be imbalanced. The majority of OPC tend to be increasingly associated with HPV infection and hence more favorable local control (22). In our dataset, HPV-negative and locally recurrent OPC only constituted 14.9 and 7.6% of the overall cohort, respectively.

Moreover, the enormous number of potential predictor variables used in radiomics studies necessitates the use of large-scale datasets in order to overcome barriers to statistical inference (23). The dearth of such datasets hinders machine-learning innovation in radiation oncology by restricting the pool of innovation to the few institutions with the patient volume to generate usable datasets (24).

#### Data anonymization

The PHI anonymization software we applied was cumbersome, requiring PHI tags to be manually entered on an individual basis. For future radiomics challenges, we recommend the use of the Clinical Trial Processor (CTP), developed by the Radiological Society of North America (RSNA) (25). Safe, efficient, and compatible with all commercially available picture archiving and communication systems (PACS), RSNA CTP is designed to transport images to online data repositories (25). RSNA CTP conforms closely to image anonymization regulations per the HIPAA Privacy Rule and the DICOM Working Group 18 Supplement 142 (16).

#### Data curation and standardization

Standardization and harmonization of data attributes provide the foundation for developing comparable data among registries that can then be combined for multi-institutional studies (26). This further empowers validation studies and subsequent generalization of the resulting models from such studies. In our challenges, VOIs were not consistently coded across the whole cohort, according to one contestant, a finding necessitating our correction to facilitate subsequent analysis for contestants.

Hence, we recommend conforming to common ontology guidelines when assigning nomenclature for target volumes and clinical data. Good examples would be the American Association of Physicists in America Task Group 263 (AAPM TG-263) (27) and North American Association of Central Cancer Registries (NAACCR) guidelines (28).

#### Volume of interest definition and delineation

Another cumbersome aspect of data curation is the segmentation of target volumes. Reliable semi-automated segmentation methods for head and neck carcinomas and normal tissues are currently still under investigation, so we relied on manual segmentation (29, 30). The disadvantages of manual segmentation relate not only to being time-consuming but also to intra- and inter-observer variability (31). A collateral benefit of making CT datasets with expert manual segmentations publicly available is testing semi-automated segmentation tools (32).

In our case, 2 radiation oncologists were blinded to relevant clinical data and outcomes, and their segmentations were cross-checked then double-checked by a single expert radiation oncologist, to diminish inter-observer variability. Guidelines of the International Commission on Radiation Units and Measurements reports 50 and 62 were followed when defining target volumes (33, 34).

#### Scanner and imaging parameters variability

Variability in inter-scanner and imaging acquisition parameters, like voxel size, reconstruction kernel, tube current and voltage has been shown to influence radiomics analyses (35–39). Thus, when sharing imaging data with contestants and uploading to public data repositories, we recommend preserving all DICOM headers aside from those containing protected health information. These parameters, easily extractable from DICOM headers, can also be provided as Supplementary Materials for future radiomics challenges. Although we did not elicit specific feedback in the post-challenge survey regarding how contestants accounted for differences in image acquisition, we recognize the importance of this question and recommend its inclusion in future radiomics challenge contestant surveys.

Moreover, head and neck radiomics are subject to the effects of image artifacts from intrinsic patient factors, such as metal dental implants and bone. The effects of resulting streak artifacts and beam-hardening artifacts on robustness of extracted radiomics features have been reported (3, 40). Our approach within this study was to remove slices of the GTV on computed tomography that were affected by artifacts. However, this results in missing information or contours that do not adequately represent the primary tumor lesion, as was noted by some contestants.

Single-institutional radiomics databases like the one used in our challenges minimize inter-scanner variability. However, in some cases the increased heterogeneity of multi-institutional databases is preferred. The choice (i.e., single vs. multi-institutional data) should be challenge-dependent. Single-institutional data may be preferred if uniformity in some imaging characteristics (e.g., slice thickness, acquisition protocol) is required for exploratory research purposes. Multi-institutional data are preferred as the end goal of radiomics challenges and studies is to generate clinically relevant models with maximum generalizability to other patient populations.

#### Interplay between clinical and radiomics data variables

We sought to provide the option to include not only physical variables but also key clinical attributes in the model building. We aimed to test the capacity of radiomics features, alone or in combination with clinical features, to model classification or risk prediction scenarios. Interestingly, the winner of the “local recurrence” challenge and the winner of the “HPV” challenge used only clinical and only radiomics data, respectively. Ironically, the fact that some contestants could generate more effective non-radiomics models for risk prediction may subvert the entire aim of the challenge. This in turn demonstrates the difficulty of integrating radiomics into clinical data in both challenge and clinical settings.

In the OPC setting, we recommend that HPV status be provided for all cases, being an independent prognostic and predictive biomarker in the OPC disease process (17, 41). However, it is also important for future radiomics challenges to consider whether other clinically relevant factors like smoking history, tumor subsite, or race are pertinent to the end goal of their challenge.

#### Quality assurance

It is important for quality assurance measures used in radiomics challenges to mirror those of traditional radiomics studies. If the dataset has not been used in a radiomics analyses, it is imperative for test analyses to identify errors. Although we had quality assurance protocols in place, contestants still noted issues with the dataset. Using Kaggle in Class, contestants were able to report feedback in real time. In turn, the responses we posted to the Kaggle in Class Forum could be viewed by all groups, ensuring that all contestants had access to the same updated information at all times, regardless of who originally asked a question. As the challenge progressed, contestants reported 9 corrupt, inaccessible DICOM imaging files and 18 patients with GTVps which did not adequately encompass the primary gross tumor volume. In other cases, the GTVp contours were absent, meaning these patients only had GTVn contours—the use of which was prohibited by challenges rules. Although we responded to contestant feedback in real time, we believe that clear and explicitly stated challenge rules as well as an initial test run of the data are essential.

#### Recruiting contestants

Participation in the radiomics challenges by academic groups with radiomics expertise was lower than anticipated. This reticence may be due to the public nature of the challenge combined with the uncertainty of success inherent in analyzing new datasets in limited timeframes, as well as the lack of clear translation to publishable output. An alternative explanation is that machine learning challenges platforms like Kaggle in Class are less well known to the radiomics community in comparison to the MICCAI community.

To attract contestants with radiomics expertise, it is necessary to ensure proper incentives are in place. Challenge announcements should be made well in advance of the challenge start date to provide sufficient time for contestants to include the challenge into their work plans. Partnering with renown organizations like NCI QIN and MICCAI on the challenge provides institutional branding which may draw in academic groups. Offers of co-authorship on future publications stemming from the challenge, as well as seats on conference panels at which challenge results will be shared, may boost participation.

Email distribution lists of professional societies such as MICCAI, SPIE (The International Society for optics and photonics) Medical Imaging, and The Cancer Imaging Archive (TCIA) would be an effective way to reach academics. Platforms like Kaggle and KDnuggets are more popular among non-academics interested in machine learning challenges.

#### Understanding contestant preferences

Contestants in our challenge wished to have additional data beyond what was provided. For instance, multiple contestants noted that some patients had missing VOIs on certain slices of the image. We had made the choice to omit these slices because the VOI in these regions was significantly obscured by dental artifact. However, contestants felt that shape and spatially-derived features might be affected by omission of these slices. To avoid this situation in future radiomics challenges, we suggest providing two datasets, one with artifacts included and one with artifacts excluded. This arrangement allows contestants the choice of which dataset to analyze.

#### Public and private leaderboards

The problem of overfitting has been observed in previous radiomics studies (7). Blinding contestants to their model's performance on the private test subset ensured that contestants were not overfitting their data to the test set. Hence, we chose the Kaggle in Class platform to host the challenges because it offers both public and private leaderboards based on public and held-out subsets of the test dataset, respectively. This design choice appeared to serve its intended purpose. In the “HPV” challenge, the first-place team on the public leaderboard had an AUC of 1.0 but finished in last place on the private leaderboard with an AUC of 0.52. This discrepancy suggests that their model suffered from overfitting issues. In contrast, the winner of the “HPV” challenge performed well on both public and private leaderboards, indicating that their proposed model was more generalizable.

#### Clarity of challenges rules

One difficulty inherent in radiomics challenges is variability in interpretation of challenge rules. This variability may be driven by differences in contestants' technical expertise, culture, background, and experiences. Thus, clear and unambiguous rules and challenge design are desirable. For example, our challenge rules clearly stated that radiomics features should be exclusively extracted from GTVp. However, GTVp was unavailable for some patients, typically post-surgical patients with no available pre-treatment imaging. When combined with the fact that we also provided GTVn for all patients, some contestants were confused by the conflicting messages they received. Thus, to prevent confusion it is important that the stated rules of the challenge be consistent with all other aspects of the contestants' experience during the challenge.

Furthermore, while the challenges were branded as “radiomics challenges,” we allowed the submission of models based solely on clinical prognostic factors, as was the case for the winner of the “local recurrence” challenge. In some instances, a clinical-only model may be useful as a comparison tool to determine whether there is an incremental benefit to leveraging radiomics data compared to clinical-only models. However, the permissibility of clinical-only models in radiomics challenges must be stated explicitly in contest rules to prevent confusion.

#### Collecting contestants' feedback

Another learning point relates to increasing post-contest survey response rates. A mere 50% of contestants responded to our post-challenge survey. To ensure a high survey response rate, we suggest including a pre-challenge agreement in which contestants pledge to complete the post-challenge survey as part of the challenge. A manuscript co-authorship contingent upon survey participation might also incentivize more contestants to fill out the survey.

#### Contestants' responsibilities

Participation in radiomics challenges necessitates a good-faith commitment on the part of contestants to follow through with the challenge, even in the face of unsatisfactory model performance. Withdrawals are antithetical to the mission of radiomics challenges as a learning tool for both challenge contestants and organizers to advance the field.

#### Permanent data repositories

The decision to upload our dataset to an online data repository, in this case figshare (https://doi.org/10.6084/m9.figshare.c.3757403.v1 and https://doi.org/10.6084/m9.figshare.c.3757385.v1) (18, 19), was not difficult. This was done to provide a curated OPC database for future radiomics validation studies. Furthermore, all contestants who downloaded the database during the challenge would already have access to the data, and it would have been impractical to ask all contestants to delete this information once downloaded.

We are also in the process of uploading this dataset as a part of a larger matched clinical/imaging dataset to TCIA. Versioning, which is a built-in feature in most data repositories including figshare, is essential for updating datasets, e.g., following quality assurance as well as retrieving previous versions later. To date, we have received multiple requests to use our dataset for external validation of pre-existing models.

We chose not to make available the “ground truth” of the private test subset data. The decision to withhold this information diminishes the overall value of the database to researchers using the dataset but in return preserves these test cases for future challenges.

#### Post-challenge methodology and results dissemination

One potential obstacle to disseminating radiomics challenge results relates to participant requests for anonymity. A participant's right, or lack thereof, to remain anonymous in subsequent publications of challenge results must be stated prior to the start of the challenge. Anonymity poses issues with reporting methodologies and subsequent model performance results, as these results may be traceable to the original online Kaggle in Class challenge website, where identities are not necessarily obscured. Transparency of identities, methodologies, and results is in the spirit of data sharing and is our preferred arrangement in radiomics challenges.

Scientific papers analyzing the individual performances of winning algorithms submitted to the Challenge, along with database descriptor have been or will be published (14, 21). In general, we also recommend publishing a post-challenge data descriptor that details data configuration as a guide for future dataset usage (14).

### Conclusions and future outlook

In summary, the MICCAI 2016 radiomics challenges yielded valuable insights into the potential for radiomics to be used in clinically relevant prediction and classification questions in OPC. Furthermore, our experience designing and executing the radiomics challenge imparted lessons which we hope can be applied to the organization of future radiomics challenges, such as those associated with the MICCAI 2018 Conference.

## Data availability statement

Datasets are in a publicly accessible repository: The datasets generated for this study can be found in figshare; https://doi.org/10.6084/m9.figshare.c.3757403.v1 and https://doi.org/10.6084/m9.figshare.c.3757385.v1.

## Author contributions

Substantial contributions to the conception or design of the work; or the acquisition, analysis, or interpretation of data for the work; Drafting the work or revising it critically for important intellectual content; Final approval of the version to be published; Agreement to be accountable for all aspects of the work in ensuring that questions related to the accuracy or integrity of any part of the work are appropriately investigated and resolved. Specific additional individual cooperative effort contributions to study/manuscript design/execution/interpretation, in addition to all criteria above are listed as follows: HE manuscript writing, direct oversight of all image segmentation, clinical data workflows, direct oversight of trainee personnel (ALW, JZ, AJW, JB, SA, BW, JA, and SP). TAL, SV, and PY wrote sections of the manuscript. AM primary investigator; conceived, coordinated, and directed all study activities, responsible for data collection, project integrity, manuscript content and editorial oversight and correspondence. AK, ALW, JZ, AJW, JB, SC, and SP clinical data curation, data transfer, supervised statistical analysis, graphic construction, supervision of DICOM-RT analytic workflows and initial contouring. SA, BW, JA, and LC electronic medical record screening, automated case identification, data extraction, clinical. Participated in at least one radiomics challenge, submitted a valid results and completed post-challenge questionnaire (KN, RG, CC, XF, CS, GP, SM, RL, CH, KY, TL, VB, JS, AG, AP, HP, VG, GC, GM, DV, SL, DM, LEC, JF, KF, JK, CF).

### Conflict of interest statement

The authors declare that the research was conducted in the absence of any commercial or financial relationships that could be construed as a potential conflict of interest.
