# Supplementary material for: Machine Learning Applications in Head and Neck Radiation Oncology: Lessons From Open-Source Radiomics Challenges
Source: Front Oncol. 2018 Aug 17;8:294. doi: 10.3389/fonc.2018.00294 (PMC6107800; doi:10.3389/fonc.2018.00294)
Supplement: Supplementary file 1 [file Table_1.docx]

Supplementary Material

Review Article

**Machine Learning Applications in Head and Neck Radiation Oncology: Lessons from Open-Source Radiomics Challenges**

MICCAI/M.D. Anderson Cancer Center Head and Neck Quantitative Imaging Working Group

Hesham Elhalawani^¥*^, Timothy A. Lin^¥^, Stefania Volpe , Abdallah S.R. Mohamed, Aubrey L. White, James Zafereo, Andrew J. Wong, Joel E. Berends, Shady AboHashem, Bowman Williams, Jeremy M. Aymard, Aasheesh Kanwar, Subha Perni, Crosby D. Rock, Luke Cooksey, Shauna Campbell, Pei Yang, Khahn Nguyen, Rachel B. Ger, Carlos E. Cardenas, Xenia J. Fave, Carlo Sansone, Gabriele Piantadosi, Stefano Marrone, Rongjie Liu, Chao Huang, Kaixian Yu, Tengfei Li, Yang Yu, Youyi Zhang, Hongtu Zhu, Jeffrey S. Morris, Veerabhadran Baladandayuthapani, John W. Shumway, Alakonanda Ghosh, Andrei Pöhlmann, Hady Ahmady Phoulady, Vibhas Goyal, Guadalupe Canahuate, G. Elisabeta Marai, David Vock, Stephen Y. Lai, Dennis S. Mackin, Laurence E. Court, John Freymann, Keyvan Farahani, Jayashree Kalpathy-Cramer and Clifton D. Fuller^*^

Running title: Radiomics Challenges in Radiation Oncology

^¥^ Both authors contributed equally to this manuscript

*Correspondence: **Hesham Elhalawani (**[**hmelhalawani@mdanderson.org**](mailto:hmelhalawani@mdanderson.org)**); Clifton D. Fuller (**[**cdfuller@mdanderson.org**](mailto:cdfuller@mdanderson.org)**)**

**Supplementary Table 1.** Detailed responses of some contestants to post-challenges surveys distributed by the organizers

| Team # | Challenge Entered (HPV and/or Local Recurrence) | Nationality | Academic vs. Non-Academic | Affiliation Name | Professional Domain | # Team Members | Age of Team Lead | Hours Dedicated per Team Member | Data Type(s) Used in Predictive Model | Software Used |
| --- | --- | --- | --- | --- | --- | --- | --- | --- | --- | --- |
| 1 | Local Recurrence | Italy | Academic | Universita degli Studi di Napoli | Pattern Recognition, Biomedical Image Processing, Computer Security, Biometrics, Digital Image Forensics | 4 | 47 | 30 | Clinical + Radiomics | Matlab, WEKA |
| 2 | HPV only | USA | Academic | MDACC | Biostatistics | 7 | 28 | 10 | Radiomics | IBEX |
| 3 | Both | USA | Academic | MDACC | Medical Physics | 3 | 30 | 20 | Radiomics | IBEX, R, Matlab |
| 4 | Both | USA | Academic | MDACC | Radiation Oncology | 2 | 28 | 10 | Clinical | IBEX |
| 5 | Both | USA | Academic | Colgate University | Mathematics/ Statistics | 1 | 22 | 20 | Clinical | R |
| 6 | Both | Germany | Academic | - | Psychology/ Computer science | 1 | 26 | 15 | Clinical | R |
| 7 | Both | USA | Academic | University of South Florida | Computer science/ Engineering | 4 | 33 | 25 | Radiomics | Matlab |
| 8 | Local Recurrence | India | Academic | IIT Hyderabad | Computer Science |  | 20 | 6 | Clinical + Radiomics | Python, R |

| Team # | Machine Learning Technique |
| --- | --- |
| 1 | Random Forest (Java coded) in WEKA (Waikato Environment for Knowledge Analysis) with class balancing approach. |
| 2 | Ensured training and test datasets similarity in terms of feature quality. Features were selected if differentially distributed between patients with local tumor recurrence vs. patients without local tumor recurrence in the training dataset. The correlation between these differentially distributed features and the responses (i.e. recurrence and no recurrence) were then calculated to select candidate features. Last, the features were ranked by model performance when built in a model with the feature alone. Final predictive model was chosen via a forward selection method. |
| 3 | Classification schemes |
| 4 | Predictive models: Neural nets, Support Vector machines. For the local tumor recurrence challenge: used logistic regression with gradient descent and achieved a score of .93038 (if I had picked this, it would have changed our ranking to first place). For the HPV prediction competition: used a neural network and achieved a score of .81103. |
| 5 | Logistic regression using extreme gradient boosting trees |
| 6 | Lasso regression |
| 7 | Combination of classifiers (e.g. random forests, naive Bayes, ACM) and boosting algorithms (e.g. AdaBoost). Also, oversampling techniques (e.g. SMOTE). |
| 8 | Decision Trees |

| Team # | Statistical Approach |
| --- | --- |
| 1 | AUC maximization in Leave-One-Out Cross Validation |
| 2 | First, Wilcoxon rank sum test was conducted between training and test dataset features, we only kept the ones with p-­values > 0.2 (I had to check the histogram of the boundary features to determine whether or not to include the feature and then set up the cut off). Then, on training dataset, between LRP and no recurrence subjects, Wilcoxon rank sum test was performed, and the features with p­-values less than 0.05 were kept. The correlation was then calculated between the remaining features and the response, discarding the features with extremely low correlation (e.g. less than 0.05 in absolute value). The selected features were ranked by their performance when they were fed to build the model by themselves. Here we used a random split strategy where we randomly picked 90% of the data as training and held 10% out as testing data. The random splitting was done 10 times, and the average AUC was used as the ranking reference. The model was built using a forward selection strategy, where the predictors were included in the model one by one following their rank, and addition of features stopped when the AIC of the current model stopped improving. |
| 3 | Using sparse matrices, we tried Support Vector Machine, AdaBoost, random forests, etc. |
| 4 | As previously described in |
| 5 | N.A. |
| 6 | N.A. |
| 7 | N.A. |
| 8 | N.A. |

| Team # | Parameters Used to Build Predictive Model |
| --- | --- |
| 1 | Ensemble of Random Forest (100 x Decision Trees per each Random Forest) to deal with class imbalancing. The Number of Random Forest is congruent with the majority class size. Weighted majority voting to combine results. |
| 2 | Pdfcluster package in R. The main parameter in pdfcluster is "no.stage", which was set to be 5 |
| 3 | N.A. |
| 4 | For the logistic regression with gradient descent I used 100 iterations and regularization with a parameter value of 0.1. For the neural network, I used 1 hidden layer with 100 nodes, regularization with a parameter of 1 and 1000 iterations. I used back propagation to calculate partial derivatives in order to run gradient descent. |
| 5 | Given features, processed categorical features and gradient boosting trees parameters |
| 6 | Lambda |
| 7 | There were several parameters involved with each such as 200 trees for random forests, etc. |
| 8 | N.A. |

| Team # | Feature(s) Most Relevant to Predictive Model |
| --- | --- |
| 1 | Euclidean distance (in mm - w.r.t. the centroids) between the primary tumor and lymph nodes (min, max, mean, std); Primary tumor volume (cm^3); Primary tumor longest and shortest radius (mm - w.r.t. the centroid of the tumor) |
| 2 | Maximum intensity in each ROI, and volume of the convex hull of the 3D ROI |
| 3 | Shape features |
| 4 | Gross tumor characteristics |
| 5 | - |
| 6 | Clinical data: Smoking_Pack.Years , Tumor_subsiteSoft_palate , T_category2, N_category2c , KM_Overall_survival_censor1 |
| 7 | Texture features, such as GLCM and LBP |
| 8 | N.A. |

| Team # | Obstacles Experienced |
| --- | --- |
| 1 | Some ROIs were not correctly coded: in some patients ROI information were not present for a few slice intervals; in some patients there was no information about the primary tumor (only the lymph nodes were depicted). |
| 2 | We couldn't find a way to extract global directional features; thus, in our implementation, any feature that has a direction restriction was not considered. |
| 3 | Image issues. Couldn't get radiomic features for all patients |
| 4 | Data dropout. I wasn't able to extract radiomics features for all images |
| 5 | Pixels are mostly zero |
| 6 | Missing values |
| 7 | Registering segmentation files with CT images (some segmentation files seemed to be out of the volumes) |
| 8 | N.A. |
