# Supplementary material for: Machine Learning Applications in Head and Neck Radiation Oncology: Lessons From Open-Source Radiomics Challenges
Source: Front Oncol. 2018 Aug 17;8:294. doi: 10.3389/fonc.2018.00294 (PMC6107800; doi:10.3389/fonc.2018.00294)
Supplement: Supplementary file 3 [file Table_3.docx]

Supplementary Material

Review Article

**Machine Learning Applications in Head and Neck Radiation Oncology: Lessons from Open-Source Radiomics Challenges**

MICCAI/M.D. Anderson Cancer Center Head and Neck Quantitative Imaging Working Group

Hesham Elhalawani^¥*^, Timothy A. Lin^¥^, Stefania Volpe , Abdallah S.R. Mohamed, Aubrey L. White, James Zafereo, Andrew J. Wong, Joel E. Berends, Shady AboHashem, Bowman Williams, Jeremy M. Aymard, Aasheesh Kanwar, Subha Perni, Crosby D. Rock, Luke Cooksey, Shauna Campbell, Pei Yang, Khahn Nguyen, Rachel B. Ger, Carlos E. Cardenas, Xenia J. Fave, Carlo Sansone, Gabriele Piantadosi, Stefano Marrone, Rongjie Liu, Chao Huang, Kaixian Yu, Tengfei Li, Yang Yu, Youyi Zhang, Hongtu Zhu, Jeffrey S. Morris, Veerabhadran Baladandayuthapani, John W. Shumway, Alakonanda Ghosh, Andrei Pöhlmann, Hady Ahmady Phoulady, Vibhas Goyal, Guadalupe Canahuate, G. Elisabeta Marai, David Vock, Stephen Y. Lai, Dennis S. Mackin, Laurence E. Court, John Freymann, Keyvan Farahani, Jayashree Kalpathy-Cramer and Clifton D. Fuller^*^

Running title: Radiomics Challenges in Radiation Oncology

^¥^ Both authors contributed equally to this manuscript

*Correspondence: **Hesham Elhalawani (**[**hmelhalawani@mdanderson.org**](mailto:hmelhalawani@mdanderson.org)**); Clifton D. Fuller (**[**cdfuller@mdanderson.org**](mailto:cdfuller@mdanderson.org)**)**

**Supplementary Table 3.** Receiver operating characteristic area under the curve (AUC) of contestants’ models and corresponding teams ranking for “local recurrence” challenge

| Private leaderboard ranking | Team # | Best Public AUC | Best Private AUC |
| --- | --- | --- | --- |
| 1 | 5 | 0.740 | 0.924 |
| 2 | 9 | 0.806 | 0.919 |
| 3 | 6 | 0.814 | 0.905 |
| 4 | 4 | 0.690 | 0.867 |
| 5 | 10 | 0.841 | 0.805 |
| 6 | 7 | 0.647 | 0.712 |
| 7 | 2 | 0.600 | 0.696 |
| 8 | 12 | 0.598 | 0.687 |
| 9 | 13 | 0.640 | 0.674 |
| 10 | 14 | 0.544 | 0.668 |
| 11 | 15 | 0.689 | 0.500 |
| 12 | 3 | 0.500 | 0.500 |
| 13 | 16 | 0.500 | 0.500 |
| 14 | 8 | 0.473 | 0.449 |
